# Monitoring Effect of Fire on Ant Assemblages in Brazilian Rupestrian Grasslands: Contrasting Effects on Ground and Arboreal Fauna

**DOI:** 10.3390/insects8030064

**Published:** 2017-06-23

**Authors:** Diego Anjos, Ricardo Campos, Renata Campos, Sérvio Ribeiro

**Affiliations:** 1 Laboratory of Evolutionary Ecology of Canopy Insects and Natural Succession, Department of Biodiversity, Evolution and Environment, Federal University of Ouro Preto, Ouro Preto 35400-000, MG, Brazil; rbfcampos@gmail.com (R.C.); spriberio@iceb.ufop.br (S.R.); 2 Postgraduate Program in Entomology, Faculty of Philosophy, Sciences and Letters of Ribeirão Preto, University of São Paulo, Ribeirão Preto 14040-901, SP, Brazil; 3 Postgraduate Program in Ecology, Department of General Biology, Federal University of Viçosa, Viçosa 36570-900, MG, Brazil; ricardo.campos@ufv.br; 4 Postgraduate Program in Integrated Management of the Territory, University of Vale do Rio Doce, Governador Valadares 35030-260, MG, Brazil

**Keywords:** spatial segregation, fire policy, community structure, insects, burn

## Abstract

Fire is one of the most relevant ecological disturbances in nature. Little is known about the effects of fire on biodiversity in ecosystems like rupestrian grasslands, which share characteristics with savanna and forest biomes. Brazilian rupestrian grasslands are part of an endangered ecosystem that has been modified by anthropogenic fire events that have become more intense in recent decades. In this study, we evaluated the effects of fire on ground and arboreal ant assemblages through a two-year monitoring program (24 monthly samplings). We found that fire does not change cumulative species richness after 24 months, and that fire does not affect mean ant richness, abundance, and species composition in arboreal ants. On the other hand, fire increased mean ground ant species richness and abundance, and caused a significant change in species composition. Our results indicate a weak and beneficial effect of fire only for ground ant communities, which generally agrees with results from other studies in Brazilian savannas. Taken together, results from these studies may be useful for improvement of fire suppression policy in fire-prone habitats in Brazil.

## 1. Introduction

Brazilian rupestrian grasslands are considered one of the most diverse rocky outcrop formations in the world [1], containing a high number of endemic species and presenting a mosaic of vegetation types ranging from open grasslands to forest formations [2,3,4]. Rupestrian grasslands are commonly affected by fire and the natural fire regime is important for breaking seed dormancy [5], changes in productivity, and nutrient content of post-fire regrowth and plant flowering [6]. However, due to the increase of human activities (e.g., agriculture and cattle grazing) the frequency and intensity of anthropogenic fire has increased in rupestrian grasslands, especially in the last five decades [1,7,8]. Fire is considered one of the most important ecological disturbances due to its potential to generate significant changes in species diversity and ecosystem functioning [9,10]. Nevertheless, very little is known about the consequences of fire on species biodiversity in rupestrian grasslands [11] and this is especially worrying due to present fire suppression policy in Brazil, which basically mandates zero tolerance with burning events [1].

With respect to the impacts of fire, ants are one of the best-studied animal taxa [12,13,14,15,16]. In fire-prone habitats—such as the tropical savannas of Australia, Africa, and South America—the natural regime (higher frequency and lower intensity) can increase the structural diversity of vegetation, generating long-term positive effects on ant diversity [14,17,18]. On the other hand, forest studies have shown strong and negative consequences of fire (e.g., fire from human activities) on ant diversity at all spatial and temporal scales [19,20,21]. With regards to the rupestrian grasslands, there is little information about effects of fire on ant communities (but see [15,16]). Our expectation is that ant community responses to fire are likely more similar to those typical of savanna than to forest habitats [16], as rupestrian grasslands possess floristic and landscape features that are more similar to Brazilian savanna than Atlantic forest [3].

The ground ants are as diverse as arboreal species. Yet, contrasting effects of fire are expected for these two habitat strata [12]. Temperature is not significantly increased by fire from 5 cm below ground in savannas [22,23]. Thus, fire has a smaller direct effect on ground-nesting ants compared to arboreal fauna. However, intense fire events have the capacity to destroy arboreal ant nests in the Brazilian savanna [24]. Even when the trees get just singed the sharp increase in air temperature caused by fire can kill whole arborous ant nests. Moreover, six months after a fire event, mean species richness increased for ground ants and decreased for arboreal ants in the Brazilian savanna [12]. Nevertheless, to our knowledge, no studies have tested the effects of fire on ant fauna comparing ground and arboreal species in Rupestrian grasslands.

This study evaluated the effects of fire on ground and arboreal ant assembly structure in a rupestrian grassland, through monthly sampling over two years. Our general aim was determine if the effects of fire on ant assemblages in rupestrian grasslands should respond more similarly to Brazilian savanna than forest communities, generating a positive effect on ant biodiversity. For these reasons, we tested the following hypotheses: (i) fire does not change cumulative species richness over two years; (ii) fire alters ant habitat components, such as foraging habits and nesting sites, among others causing mean species richness and abundance to increase for ground ants and decrease for arboreal ants; (iii) thus, ant species composition for both ground and arboreal fauna are affected by fire.

## 2. Material and Methods

### 2.1. Study Area

The study was conducted in the Itacolomi State Park (PEIT/IEF) (20°26′26′′ S, 43°30′52′′ W), located in the towns of Ouro Preto and Mariana in Minas Gerais state, Brazil. The study area was described as a ‘rupestrian complex’ [25]. The region belongs to Itacolomi montane group, consisting mainly of quartzite, quartz, phyllite, and metaconglomerate soils, with an average altitude of 1500 m. Annual precipitation ranges from 1100 to 1800 mm, and average temperature is between 17.4 °C and 19.8 °C. The rainy season extends from October to April, and the dry season extends from May to September [26].

### 2.2. Sample Areas and Fire History

We selected two sample sites (300 m away from each other) within the park with typical rupestrian grassland vegetation. The sites had similar vegetation structure and the same soil type (quartzite), with rock formations and low slope [26], the same altitude (1300 m) and area (approximately 2 ha). The woody vegetation in the study areas is dominated by “Candeial” (*Eremanthus erythropappus* and *Baccharis* sp.; both Asteraceae), all shrub-tree sized [27]. Poaceae and Asteraceae are highly abundant in the grass layer (see [27]).

The two study areas were selected according to their fire history, with one classified as “burnt area” and the other as “unburnt area”. The “burnt” area had recent records of fire (at least four major fire events in the past decade), with the most recent burning occurring four months prior to our first sampling. The “unburnt” area was protected from fire for at least 10 years prior to the present study [28].

### 2.3. Ant Sampling

Within each study site, we established one 15 m × 20 m grid (300 m^2^). Inside each grid, 20 pitfall traps were set up on the ground (space pitfalls farther 5 m apart). For arboreal ant sampling, 20 pitfall traps were established on woody vegetation. Each arboreal pitfall trap was tied to the trunk of the shrub/tree closest to the ground pitfall at approximately 1.20 m height. The pitfalls were made from 20 mL plastic containers with 3 cm diameter, and stayed open in the field for 96 h [15]. Ants were sampled monthly between October 2010 and October 2012, totaling 960 samples per grid/area. Ants were identified to the lowest possible taxonomic level and subsequently deposited in the Entomological Collection of the Federal University of Ouro Preto. All identifications of ant species were carefully reviewed by a qualified taxonomist, Dr. Rodrigo Feitosa (Federal University of Parana, Curitiba, Brazil).

### 2.4. Statistical Analysis

To compare the overall ant species richness on the ground and in trees between the burnt and unburnt areas, we generated four rarefaction curves (MaoTau method) using cumulative species richness over the 24 months of study [29]. Rarefaction curves with non-overlapping confidence intervals were considered significantly different (C.I. 95%) [30]. Rarefaction curves were generated using EstimateS 9.1.0 [31].

We used a repeated measures factorial ANOVA [32] to compare average species richness per pitfall per month (species density) along the study period. In this model, the mean number of species (richness) and individuals (abundance) in each pitfall were response variables, and sites (burnt and unburnt area) and stratum (ground vs. arboreal) were used as factors (explanatory variables). Because samples were taken repeatedly in the same locations, the time variable (month) was considered as repeated measure in the model [32]. Finally, to avoid spatial pseudoreplication (due to distance between pitfalls within the grid) the ‘grid’ was considered a random block effect in the model.

We used multivariate permutational variance (PERMANOVA) to test for changes in species composition between areas over time. In this analysis, time (month) and area (burnt vs. unburnt) were considered as factors, and species composition in each area within each month was considered the response variable. We performed two PERMANOVA models separately for ground vs. arboreal ant assemblages, using the sum of the relative abundances of each species collected in the 20 pitfalls (a single collection event) in each stratum and month (n = 24). This analysis was performed using R software, version 3.2.1 [33].

Finally, to assess whether the temporal changes in ant composition occurred at random in both areas (again separately for ground and arboreal species) we used a seriation null model analysis [33]. This model ranks the species according to presence (1) or absence (0) in samples, with results indicating whether changes after fire and over time display a gradient of non-random modification. The model then uses an algorithm proposed to create an ordination matrix with species in rows and months in columns [34]. The species collected temporally closer to recent fire event were positioned at the top of the matrix, and the species collected many months after fire are positioned at the end of the matrix. The program uses Monte Carlo simulations to create 30 random matrices from the original data, with the same number of occurrences for each taxon. The model then tests whether the patterns observed in the original data are different from the random patterns. The seriation analysis was performed in PAST version 3.01 [35].

## 3. Results

### 3.1. Cumulative Species Richness

**Figure 1:**
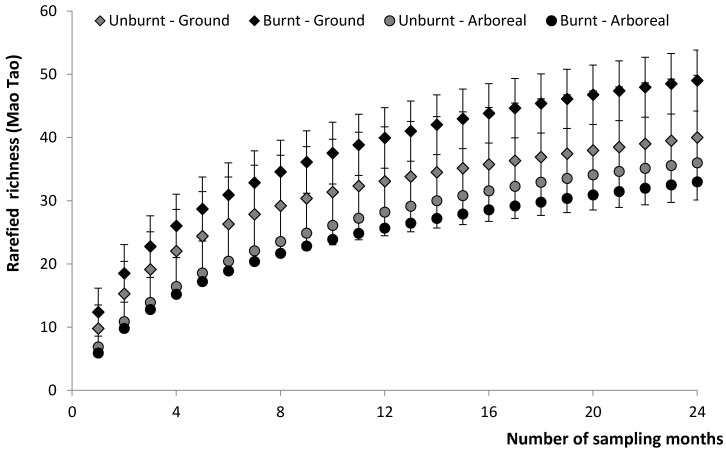
Rarefaction curves (with confidence intervals—95%) for ant species collected in the burnt area (dark symbol) and unburnt area (gray symbol). Ground ant species are represented by diamonds and arboreal ant species are represented by spheres.

We collected a total of 3459 ants belonging to 68 species, 27 genera, and 6 subfamilies. The most diverse genera were *Camponotus* and *Pheidole*, with 16 and 8 species, respectively. From the total sampling effort, 2239 ants (64.72%) were collected in the burnt area, and 1220 (35.28%) were collected in the unburnt area. The rarefaction curves showed a strong overlapping of confidence intervals, indicating that there was no difference in overall ant species richness between burnt and unburnt areas (Figure 1).

### 3.2. Ant Species Richness and Abundance Based on Mean Species Density per Sample per Month

**Figure 2:**
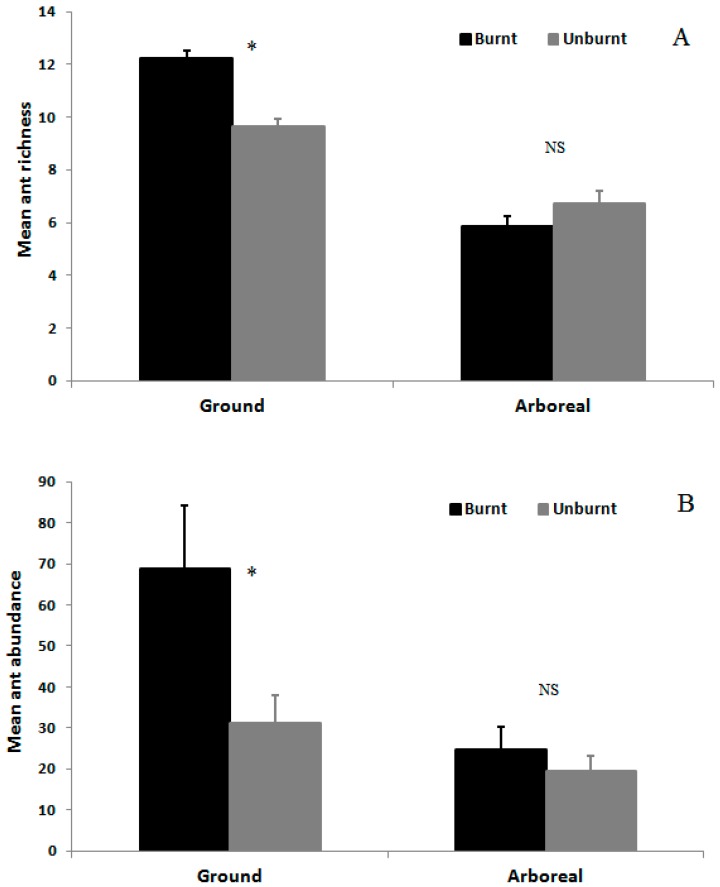
Average richness (**A**) and abundance (**B**) of ground and arboreal ant species in the burnt area (dark bars) and unburnt area (gray bars). Asterisks indicate significant differences between groups (*p* < 0.05). NS = not significant.

**Figure 3:**
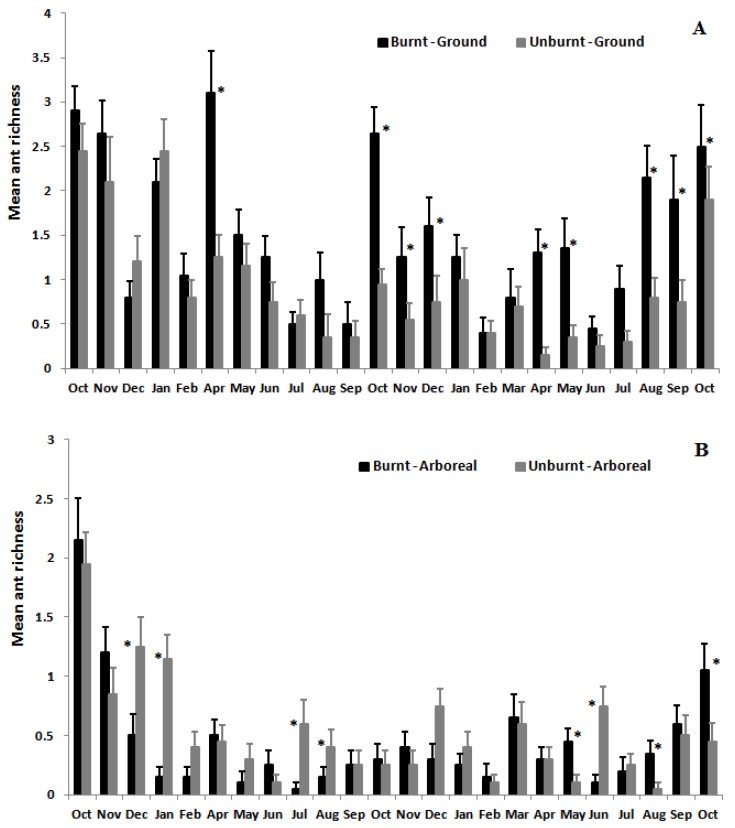
Variation in mean richness of ground ant species (**A**) and arboreal ant species (**B**) at Itacolomi State Park over two years in burnt (black bars) vs. unburnt (grey bars) areas. Asterisks indicate significant differences among months (*p* < 0.05).

**Figure 4:**
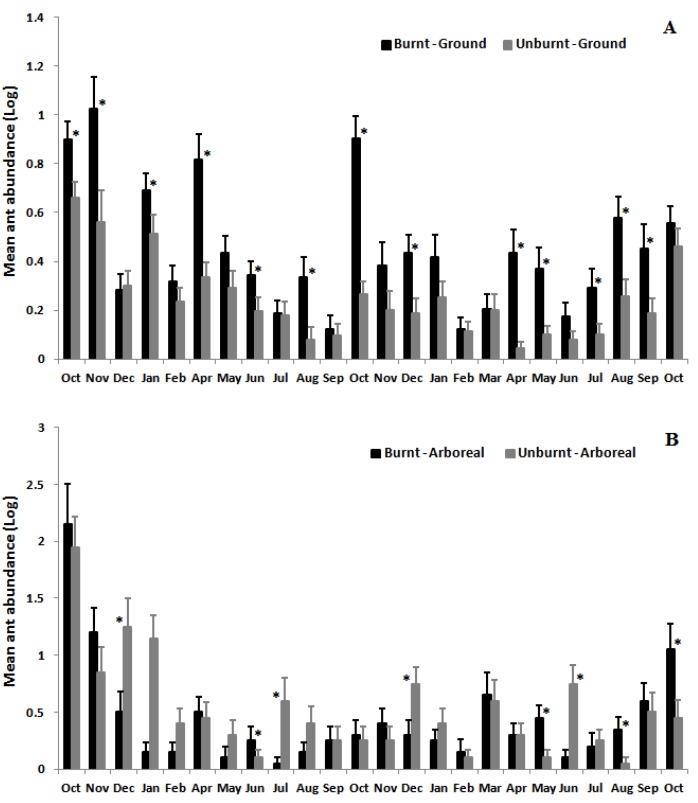
Variation in mean abundance of ground (**A**) and arboreal ant species (**B**) at Itacolomi State Park over two years in burnt (black bars) vs. unburnt (grey bars) areas. Asterisks indicate significant differences among months (*p* < 0.05).

Mean ant species richness (12.3 ± 0.3; mean ± SD) and abundance (69.1 ± 17.5; mean ± SD) on the ground were greater in the burnt area than in the unburnt area (richness: *F*_1,38_ = 36.40, *p* < 0.001; abundance: *F*_1,38_ = 57.96, *p* < 0.001) (Figure 2). However, arboreal ant species richness and abundance did not differ between burnt and unburnt areas (richness: *F*_1,38_ = 1.31, *p* = 0.26; abundance: *F*_1,38_ = 0.17, *p* = 0.68; Figure 2). There was a significant interaction between the effects of fire and time (month) on ground ants (richness: *F*_23,874_ = 2.18, *p* < 0.001) and arboreal ants (richness *F*_23,874_ = 3.44, *p* < 0.001), indicating that the effect of fire on mean ant species richness varied temporally (Figure 3). Mean species richness of ground ants was higher in the burnt than in the unburnt area in 9 out of 24 months, and mean abundance was higher in the burnt area in 13 out of 24 months (Figure 3 and Figure 4). However, these differences between month in species richness were not concentrated in initial or final months of sampling. Furthermore, the same pattern was not found for arboreal fauna. Mean species richness and abundance of arboreal species was higher in the unburnt area for 5 months out of the 24, and was lower for 3 and 4 months, respectively, for species richness and abundance. Finally, most of the time (16 and 15 months out of 24 for richness and abundance, respectively) this difference was not significant for arboreal ants (Figure 3 and Figure 4).

### 3.3. Ant Species Composition

**Figure 5:**
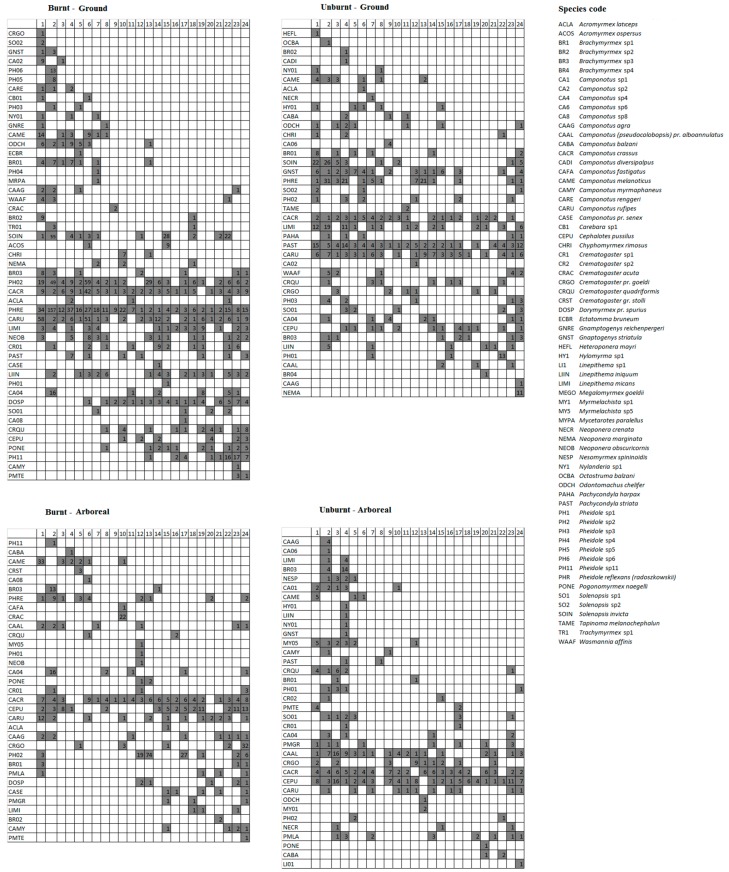
Seriation model of temporal changes in ground and arboreal ant fauna in burnt and unburnt areas. Numbers in the columns represent sample months after fire (1 to 24 months) and rows indicate ant species. Colored cells represent species presence, and the numbers within cells represent the abundance of that species for that month.

Ground ant species composition differed between burnt and unburnt areas (PERMANOVA: *F*_1,47_ = 7.85, R^2^ = 0.13, *p* < 0.001), and composition changed over the study period (PERMANOVA: *F*_1,46_ = 3.37, R^2^ = 0.05, *p* < 0.001). The area*time interaction was also significant (*F*_1,45_ = 1.81, R^2^ = 0.03, *p* = 0.034), indicating that differences in species composition between the burnt and unburnt areas varied over the 24-month period. Seriation analysis showed that temporal changes in species composition both in the burnt area (Z = 1.92, *p* = 0.01) and in the unburnt area (Z = 2.64, *p* = 0.01) were not random for ground fauna (Figure 5).

For arboreal fauna, PERMANOVA also showed significant differences in species composition between areas (*F*_1,47_ = 2.77, R^2^ = 0.05, *p* = 0.002) and months (*F*_1,46_ = 2.26, R^2^ = 0.04, *p* = 0.008), however this difference did not change over time (area*time: *F*_1,45_ = 1.32, R^2^ = 0.02, *p* = 0.185). Further, low R^2^ values from PERMANOVA indicate that differences in arboreal ant species composition between burnt and unburnt areas must be taken with caution. Finally, seriation analysis showed that differences in arboreal ant species composition over time were not different from random in both burnt and unburnt areas, thus, changes in composition occurred by chance over the study period (burnt area: Z = 1.11, *p* = 0.27; unburnt area: Z = 12.49, *p* = 0.62; Figure 5).

## 4. Discussion

We found that in general the effect of fire on ant communities in rupestrian grasslands appears was weak, since it does not change cumulative species richness after 24 months, and also that fire does not affect mean ant richness, abundance, and species composition among arboreal ants. On the other hand, fire can be considered positive as it increased the mean ground ant species richness and abundance, and caused a significant change in species composition. Taking these results together, we could argue that, as expected, the effects of fire on ant community structure in rupestrian grasslands seems to be more similar to the Brazilian grasslands [14] than in forests habitats [21].

Fire had no detectable effects on cumulative ground or arboreal ant species richness over time. Although there was an apparent increase in cumulative richness over the study period for ground fauna in the burnt area (49 species) compared to the unburnt area (40 species), rarefaction analysis indicated that this difference was not significant. The absence of fire effects on cumulative species richness can be explained by several factors. First, based on the long geological time that ants have inhabited fire-prone environments such as the Brazilian savanna [12,14,36] and rupestrian grasslands [15,16], we expect these insects to be highly resistant and resilient to fire. Areas with shorter vegetation stature (e.g., rupestrian grasslands) are dominated by ants that nest in the soil, protected from direct effects of fire. This type of vegetation recovers very quickly in terms of biomass, and fire may even stimulate the growth and reproduction of many herbaceous species [6,37]. Thus, the absence of fire effects on the cumulative species richness can also be attributed to rapid natural succession of post-fire vegetation, which also decreases the indirect effects of fire on ant fauna [38,39,40].

We also found that for the majority of sampling months, mean ground ant species richness and abundance was higher in the burnt area than in the unburnt area, and that species composition was significantly different between the two areas. Over the course of two years, fire may cause direct and indirect effects on habitat that may increase ground ant activity. Generally, there is low biomass during the first few months after fire, facilitating ant foraging and consequently increasing ant activity on the ground [12]. Indeed, mean ground ant abundance was higher in the burnt than in the unburnt area in the first two months of the study (Figure 4A). Fire also destroys the leaf litter along with all food resources present within it [41], thus at least in the short term, ground ants tend to increase their foraging area for searching food [42,43]. Finally, ant foraging activity on the ground should increase due to the simple fact that before fire disturbance, they were likely foraging on low vegetation that was subsequently removed [42,43,44]. For instance, species such as *Camponotus rufipes* and *C. crassus*, which nest on the ground but often forage on low vegetation, may become more frequent on the ground after the fire, as was observed in the present study (Figure 5).

It is also important to point out that indirect positive effects for ant communities are also expected after a fire event [12]. Essential food resources such as nectar from extrafloral nectaries [45], seem to increase in availability on the scrubs after a fire event [39,40,46]. This increased resource availability can attract species from surrounding areas post-fire. This fact may partially explain the higher mean species richness and abundance of ants in the burnt area in the second year of the study (Figure 3A and Figure 4A). In another study of rupestrian grasslands in southeastern Brazil, mean species richness and abundance of ground ants increased four months after a fire event [16].

We also found evidence for greater changes in ant species composition in the burnt versus unburnt area over the 24-month period (Figure 5). There was a change of 40.8% of the species (20 out of 49) in burnt area when comparing the first and the second year of sampling, while this figure drops to 22.5% (9 out of 40) in the unburnt area. Second, the change in relative abundances of the four most frequent species on the ground was much greater in the burnt than unburnt area (*Pheidole reflexans*— (mean ± SD) burnt: 17.20 ± 31.56, unburnt: 7.66 ± 10.52; *Camponotus crassus*—burnt: 5.30 ± 8.40, unburnt: 2.00 ± 1.10; *Camponotus rufipes*—burnt: 8.23 ± 15.69, unburnt: 3.60 ± 2.52; *Pheidole* sp2—burnt: 10.65 ± 16.39, unburnt: 1.66 ± 0.81). Strong effects of fire on ant composition (i.e., changes over time) were also reported in two other studies of fire ants in rupestrian grasslands [15,16].

Despite likely effects of fire on vegetation [47,48], we did not see evidence of decreased ant foraging on plants in the burnt area. This result may be mainly attributed to the low number of tree-nesting ant species in this study (Figure 5). In fact, the most frequent and abundant species found on trees (*C. crassus* and *Pheidole* sp2) (Figure 5) probably nest in the soil and only forage on vegetation. *Camponotus crassus* was the species with greatest number of nests in natural soil cavities (see [45]), and the majority of described *Pheidole* species nest on the ground [49]. On the other hand, *Cephalotes pussilus*, which prefers nesting in trees [50,51], had decreased abundance in the first months of the study only in the burnt area (Figure 5). However, in savanna habitats where there is high diversity of tree-nesting ants [52,53,54], fire reduced the mean abundance of arboreal ant species six months after fire [12].

Despite absence of data prior to the fire event, contrasts between burnt and unburnt sites, strongly suggest that the pattern of temporal changes in ground ant species composition in the burnt area was influenced by fire. However, despite lower intensity, species composition also varied significantly over time in the unburnt area. In addition to fire, natural and unpredictable disturbances (e.g., input of animal or plant debris, the presence of army ants or other predators) may also cause changes in species composition over time in burnt areas and, to a lesser degree, in unburnt areas [15,55,56]. In this study, there appears to be a natural pattern of temporal change in ground ant species composition in both areas, and the presence of fire might intensify such effects, explaining the lower intensity of changes in species composition in the unburnt than burnt area (Figure 5).

## 5. Conclusions

This was the first study to monitor effects of fire on ground and arboreal ant communities in the highly endangered Brazilian rupestrian grassland. The effects of fire on the ant community structure were generally low in magnitude, but appear to be beneficial to ground fauna by increasing foraging activity and providing a window of opportunity for other species to occur in the fire-altered landscape. Although our findings did not differ from previous studies [15,16], they should be interpreted with caution due to lack of sampling before the fire (e.g., the possibility of previous changes in ant community structure between sampling areas cannot be excluded). However, we strongly believe that our analytical approach was robust enough to remedy this methodological limitation, and the study clearly demonstrated greater changes in community structure over time in the burnt area compared to the unburnt area. Our results indicate a weak and beneficial effect of fire on ant communities, which generally agrees with those of other studies in fire-prone habitats such as Brazilian savanna [14,41]. However, it is very important to make clear that the effect of fire on biodiversity is highly context dependent, presenting sharply contrasting results based on the taxa, frequency, and intensity of fire. Bearing it in mind, the results presented here might be helpful to better understand the impacts of fire on biodiversity and also to improve fire suppression policy in fire-prone habitats in Brazil.
